# Function and Role of Regulatory T Cells in Rheumatoid Arthritis

**DOI:** 10.3389/fimmu.2021.626193

**Published:** 2021-04-01

**Authors:** Qi Jiang, Guocan Yang, Qi Liu, Shengjun Wang, Dawei Cui

**Affiliations:** ^1^ Department of Blood Transfusion, Shaoxing People’s Hospital (Shaoxing Hospital, Zhejiang University School of Medicine), Shaoxing, China; ^2^ Department of Laboratory Medicine, The Affiliated People’s Hospital, Jiangsu University, Zhenjiang, China; ^3^ Department of Immunology, Jiangsu Key Laboratory of Laboratory Medicine, School of Medicine, Jiangsu University, Zhenjiang, China; ^4^ Department of Blood Transfusion, The First Affiliated Hospital, Zhejiang University School of Medicine, Hangzhou, China

**Keywords:** Treg cells, rheumatoid arthritis, autoimmune diseases, immune tolerance, transcription factor Foxp3

## Abstract

Rheumatoid arthritis (RA) is a systemic and heterogeneous autoimmune disease with symmetrical polyarthritis as its critical clinical manifestation. The basic cause of autoimmune diseases is the loss of tolerance to self or harmless antigens. The loss or functional deficiency of key immune cells, regulatory T (Treg) cells, has been confirmed in human autoimmune diseases. The pathogenesis of RA is complex, and the dysfunction of Tregs is one of the proposed mechanisms underlying the breakdown of self-tolerance leading to the progression of RA. Treg cells are a vital component of peripheral immune tolerance, and the transcription factor Foxp3 plays a major immunosuppressive role. Clinical treatment for RA mainly utilizes drugs to alleviate the progression of disease and relieve disease activity, and the ideal treatment strategy should be to re-induce self-tolerance before obvious tissue injury. Treg cells are one of the ideal options. This review will introduce the classification, mechanism of action, and characteristics of Treg cells in RA, which provides insights into clinical RA treatment.

## Introduction

Rheumatoid arthritis (RA) is a systemic inflammatory autoimmune disease. Musculoskeletal pain, joint swelling and stiffness are its common clinical symptoms, that seriously damage body function and reduce the quality of life of patients (1–3). Patients with RA are more likely to develop osteoporosis, infection, cardiovascular diseases, respiratory diseases, cancer and other diseases than the general population (2–4). More women than men are diagnosed with RA, and the proportion is approximately 3:1 (5). Early diagnosis, the emergence of new treatment methods, and the application of new effective treatment strategies significantly improve the long-term prognosis of the joints of patients with RA (3–6). The pathogenesis of RA is complex and includes synovial cell proliferation and fibrosis, vascular membrane formation, cartilage and bone erosion (2, 3). Naive CD4^+^ T cells can differentiate into different cells types under antigen presenting cell (APC) stimulation. An imbalance in the function and/or the number of these cells will lead to the abnormal cellular and humoral immunity (7, 8). Abnormal humoral immunity often leads to excessive activation of autoantigenic T and B cells, resulting in the abnormal production of antibodies, such as rheumatoid factor (RF) and anti-cycle citrullinated peptide (anti-CCP) antibodies, and the deposition of immune complexes in synovial tissue, resulting in persistent synovitis and joint destruction (4, 9, 10). Innate immune cells, including mast cells, dendritic cells (DCs), innate lymphocytes and adaptive immune cells, such as B cells, plasma cells, follicular regulatory T (TFR) cells and helper T (Th) cells mediate the systemic autoimmune inflammatory response (11). Abnormal activation of these cells may result in the excess production of pro-inflammatory cytokines such as IL-6, TNF and IL-17, which eventually lead to the destruction of bone tissue and cartilage (11–13). Th17 cells (T cell subsets characterized by secretion of IL-17) produce various pro-inflammatory cytokines to promote synovitis, while Treg cells inhibit inflammation and maintain immune tolerance (14–16).

## Classification of Treg Cells

Initially, Treg cells were divided into three categories according to their origin and differentiation: they are produced by immature T lymphocytes during thymus development, and with a phenotype is CD4^+^CD25^+^Foxp3^+^ T cells, which are called natural Treg (nTreg) cells that constitutively express CD25 and express the specific nuclear transcription factor Foxp3. Upon peripheral antigen stimulation or immunosuppressive factor induction, mature CD4^+^CD25^-^ T cells are transformed into acquired Treg (iTreg) cells, including Tr1 and Th3 subsets; the former mainly secretes IL-10 and TGF-β, while the latter mainly produces TGF-β. In addition to regulatory CD4^+^ T cells, regulatory CD8^+^ T cells also exist in CD8^+^ T cells (17).

Some scholars also recommend distinguishing two primary Treg cell groups according to their origin. nTreg cells are called thymus-derived Treg (tTreg) cells, which originate from the thymus and have a relatively high self-affinity T cell receptor (TCR) (18). In the periphery, CD4^+^ effector cells begin to express Foxp3, under the influence of TCR signal transduction or other factors, such as TGF-β and IL-2. These cells are called pTreg cells and are most commonly found in peripheral barrier tissues and prevent local inflammation (19). Since naive CD4^+^Tconv cells and Treg cells have non-overlapping TCR sequences, the TCR libraries of tTreg cells and pTreg cells are also quite different (20). The TCR libraries of tTreg cells are biased toward self-recognition, while the TCRs of pTreg cells identify foreign antigens with high affinity (21).

Treg cells are distributed in the T cell region of lymphoid organs and in the B cell region TFR, controlling the production and maturation of antibodies (22). According to their location, these cells have been divided into peripheral lymphoid tissue Treg cells and non-lymphoid tissue-resident Treg cells, including central (cTreg) cells and effector (eTreg) cells (23). cTreg cells account for the majority of Treg cells in secondary lymphoid organs and express CCR7 and CD62L at high levels (24), while eTreg cells express surface markers, such as ICOS or CD44 (25). Non-lymphoid tissues where Tregs have been found include visceral adipose tissue (VAT), skin, the lamina propria of the colon, lung and skeletal muscle. Tissue-specific homing receptors such as GPR15 direct Treg cells to the colon, CCR4 promotes the migration of Treg cells to the skin to control tissue homeostasis (26–28). Significant differences between Treg cells in non-lymphoid tissues and lymphoid organs have been identified. The former has a tissue-specific phenotype and is functional (29). For example, VAT-Treg cells are functional specialized tissue resident cells that depend on the transcription factor PPAR-γ, limit inflammation of the skin, intestines and central nervous system and improve the sensitivity of adipose tissue to insulin (30). According to a recent study, significant differences in the transcriptional landscape, phenotype and chromatin accessibility of VAT-Treg cells exist between sexes (31). Tissue adaptation changes occur when Treg cells transfer from lymph nodes to barrier tissue (32). These dynamic adaptations lead to the co-expression and phenotypic acquisition of transcription factors associated with other pedigrees (for example, T-bet, GATA-3, IRF-4, BCL6 or STAT3) (33).

## Immunosuppressive Mechanism of Treg Cells

**Figure 1:**
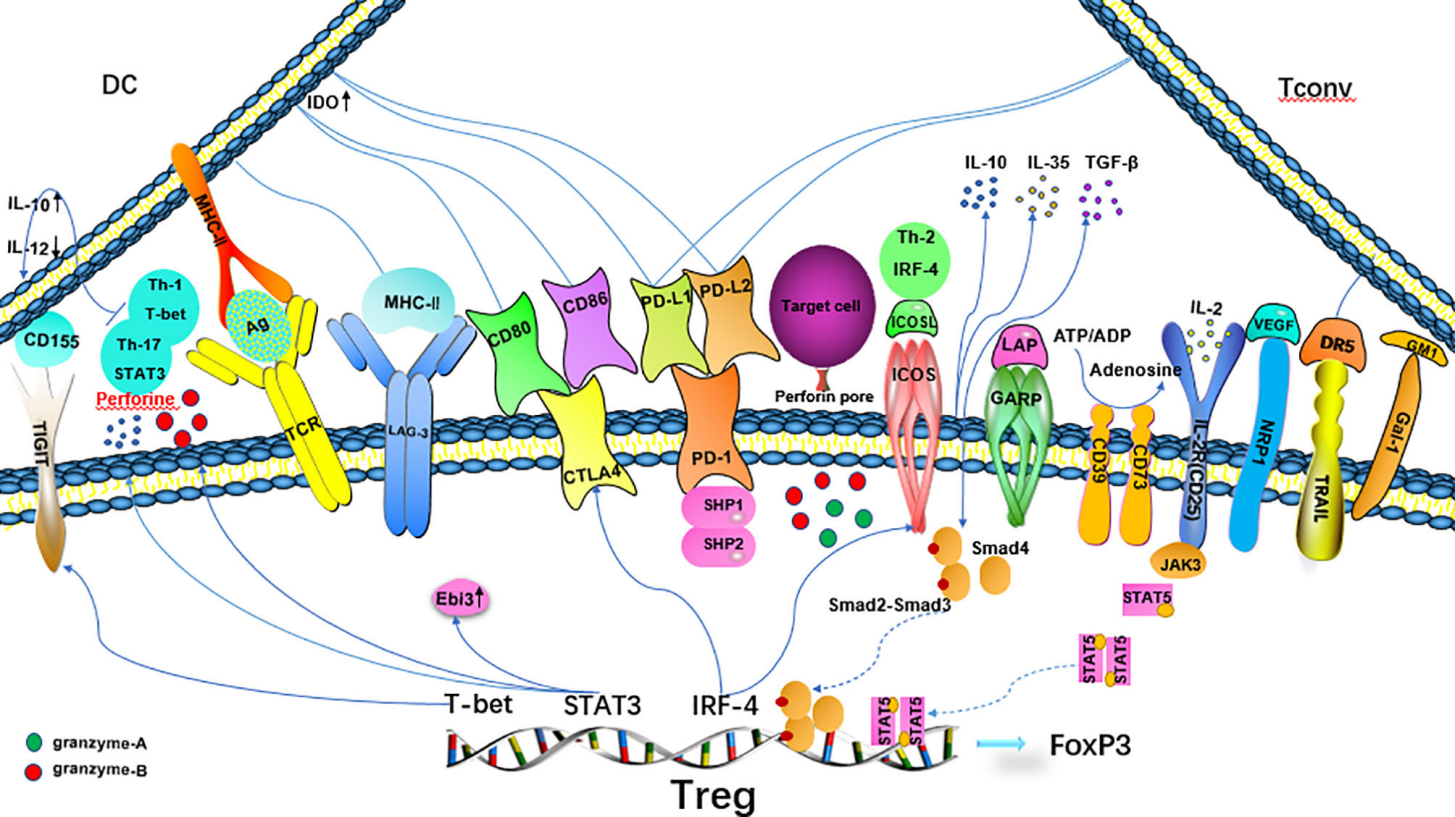
Mechanisms of Treg suppression. CTLA-4 binds to CD80/CD86 on DCs, inhibits the maturation and antigen presentation function of DCs and increases the expression of IDO in DCs, resulting in T effector incompetence. PD-1 binds to PD-L ligands on DCs and T cells to inhibit effector T cells, and synergistically enhance the transactivation of Smad3 by TGF-β.TIGIT increases IL-10 expression and decreases IL-12 expression in DCs by binding to CD155 on DCs and inhibits the activation of effector T cells. Tregs selectively inhibit the proinflammatory immune response mediated by Th1 and Th17 cells. The Th2-related transcription factor IRF-4 activates Treg expression through ICOS and CTLA-4, which restricts the Th2-mediated immune response. STAT3 increases the expression of the IL-10, Ebi3, and perforin-1 genes.

The specific inhibitory effect of Treg cells on T cells is related to the expression of these transcription factors. The ITIM domain protein (TIGIT) on the surface of Treg cells binds to CD155 on dendritic cells (DCs), resulting in an increase in IL-10 expression and a decrease in IL-12 expression in DCs, thus inhibiting the activation of effector T cells (34, 35). T-bet, a transcription factor associated with Th1 cells, is related to the expression of TIGIT. The T-bet^+^TIGIT^+^ Treg phenotype selectively inhibits the pro-inflammatory immune response mediated by Th1 and Th17 cells (36, 37). TheTh2-related transcription factor IRF-4 induces the expression of co-stimulatory molecules CTLA-4 and ICOS in Treg cells and cooperates with RBPJ and JUNB to limit the immune response mediated by Th2 cells (38–40). The expression of STAT3 (a typical Th17 transcription factor) in Treg cells is closely related to the Th17-mediated immune response, which increases the expression of the Ebi3, IL-10, and perforin-1 and granzyme B (41). Treg cells exert their inhibitory function through various mechanisms. Their mechanism of action is summarized below (**Figure 1**).

Treg cells share some surface markers with activated effector T cells, such as glucocorticoid-induced TNFR-related protein (GITR), cytotoxic T lymphocyte associated antigen-4 (CTLA-4), programmed death-1 (PD-1) and its ligand (PD-L1) (42). CTLA-4 binds to CD80 and CD86 on APCs (especially DCs), inhibits the antigen presentation and maturation function of APCs (43), and increases the expression of IDO in DCs, reducing the concentration of tryptophan necessary for effector T cell proliferation (44). PD-1 (CD279) binds to PD-L1 and PD-L2 ligands on DCs to inhibit effector T cells (45), and synergistically enhances the transactivation of Smad3 by TGF-β (46). LAG-3 binds to MHC-II, negatively regulates the function of T cells (47), and preferentially inhibits the response of T cells to the stable MHC complex (pMHC-II) (48). Neuropilin-1 (NRP1) is the receptor of vascular endothelial growth factor (VEGF). Its role differs in human and mouse, and the exact inhibitory mechanism remains to be confirmed (49). Galectin (Gal)-1 is a β-galactose-binding lectin that regulates the Treg/Th17 balance induced by DCs through the NF-κB/RelB-IL-27 pathway (50). The transmembrane protein GARP(LRRC32)/latency-related peptide (LAP) is related to the ability of Treg cells to activate TGF- β after stimulation by TCR (51). Down-regulation of GARP expression weakens the inhibitory function of Treg cells (52). TNF-related apoptosis-inducing ligand (TRAIL) is expressed when Treg cells are activated, while CD4^+^ effector cells express its ligand death receptor 5(DR5). The TRAIL/DR5 interaction activates caspase-8 to induce the apoptosis of effector lymphocytes (53, 54). CD25, also known as interleukin IL-2 receptor (IL-2R) is expressed at high levels on the surface of Treg cells. IL-2 is an important signal that induces cell proliferation *in vivo*. Treg cells compete with effector cells for IL-2 in the process of the immune response to prevent effector cells from acquiring a sufficient amount of IL-2 to proliferate (55). Treg cells exert their functions through soluble intermediates. The extracellular and/or pericellular accumulation of adenosine causes an immunosuppressive response (56). CD39/CD73 expressed on Treg cells degrade ATP into adenosine, and the increase in the adenosine concentration in the microenvironment will inhibit antigen presentation by DCs (57). The cellular lysis factors granzyme-A, granzyme-B and perforin (58, 59), anti-inflammatory cytokines IL-10, TGF-β, IL-35 and others also play a role in the immune regulation of Treg cells (60). TCR diversity was recently shown to be conducive to the expansion of Treg cells; if the specificity of TCR is the same but the affinity is different, the inhibition mechanism of Treg cells will be different (61). High-affinity receptor cells mainly express TCR-dependent mediators such as CTLA-4, GITR, IL-10 and TIGIT, in contrast, low-affinity receptor cells express more Ebi3,which is responsible for IL-35-mediated inhibition, indicating that affinity determines different inhibition mechanisms (20).

## Treg Cells in RA

An increase in the number or enhancing the inhibitory function of Treg cells may be helpful in the treatment of autoimmune diseases while reducing the number of Treg cells or inhibiting their function enhances immunity toward tumors and chronic infectious pathogens. RA is characterized by long-term chronic synovitis, cartilage necrosis, and eventually joint destruction that lead to loss of function. Many studies have recently shown that Treg cells inhibit the autoimmune response. When the number and/or function of these cells are abnormal, related antigens and DR molecules cause immune cascade amplification, which leads to the rapid increase in the levels of various cytokines in the body, such as IL-2, and activates macrophages in the synovium of bones and joints to produce many inflammatory cytokines, such as IL-1, IL-6 and IL-8. These inflammatory reactions destroy articular cartilage and eventually lead to joint deformities, leading to the occurrence of RA. However, many studies have reported contradictory results. The number of Treg cells in the peripheral blood of patients with RA is increased (62, 63), unchanged (64–66)or decreased (67–71),and contradictory results were also reported for the functional characteristics of Treg cells from RA patients, namely enhancement or attenuation (72, 73)**(** **Table 1** **)**.

**Table 1:** The frequency of Treg cells and associated cytokine levels in individuals with RA.

Class Ref.	PB	SF	IFN-γ	TNF-α	TGF-β	IL-10
62	↑	↑	*	↑	nd	*
63	↑	nd	↓	↑	nd	↑
64	—	↑	↑	↑	nd	↑
67	↓	↑	↑	↓	nd	↓
68	↓	↑	nd	↑	↓	nd

”↑” represents an increase compared to the normal control; “↓” represents a decrease compared to the normal control; “—” indicates similar to the normal control; “*” indicates a level below the detection limit; “nd” indicates not detected. PB, peripheral blood; SF, synovial fluid.

The explanation for the discrepancy is the persistent problems in the recognition of Treg cells. In most studies, the expression of Foxp3 is used to define Treg cells, but Foxp3 requires intracellular staining and the expression levels in Treg cells in the resting state and activated state are different (74). Tconv cells also express a low level of Foxp3 upon TCR stimulation (75, 76). CD25 is also a marker for activated Tconv, and Tconv also expresses low levels of CD127. Cell surface markers such as CD4^+^CD25^+/high^, CD127^low/-^, CD62 ligand, integrin Eα (CD103), GITR (TNFRSF18), CTLA-4 (CD152), CD45RO and neuropilin have been used as supplementary markers to identify Treg cells in clinical practice in addition to intracellular Foxp3 staining (77, 78). Among these markers, CD45RA and CD45RO are used to distinguish immature Treg cells (CD45RA^+^Foxp3^low^) from activated memory Treg (CD45RA^-^Foxp3^high^) cells (79). Currently, the CD3^+^CD4^+^CD25^high^CD127^low^ phenotype is most commonly isolated from Treg population through flow cytometry or immunomagnetic bead separation. The determination of inhibitory activity and demethylation of Foxp3 CNS2 are considered to be the gold standard methods for Treg identification (80, 81), specially Treg-specific DNA hypomethylation, which distinguishes Treg cells from activated Tconv cells at the genetic level. Some scholars have performed a meta-analysis on the number and proportion of Treg cells in patients with RA. The conclusion is that the use of a more stringent method to define Treg cells will reveal decreased number of Treg cells in peripheral blood and increased number in synovial fluid (82). Do Treg cells in the synovial fluid function normally?

Treg cells from patients with RA lack CTLA-4 expression in an inflammatory environment or show ineffective function due to the overexpression of IL-6 (83, 84). These Foxp3-cells are called “exTreg cells”, and a large number of exTreg cells with no inhibitory activity circulate in synovial fluid (85). However, these Treg cells show normal inhibitory activity *in vitro*, which proves that an essential disorder in these cells is not responsible for RA, rather it is caused by the inflammatory environment (67). Tregs isolated from peripheral blood might limit the proliferation of Teff cells but do not prevent the secretion of cytokines (86). Teff cells in an inflammatory environment are resistant to Treg-mediated inhibition (87). The sensitivity of CD4^+^CD25^-^ T cells and APCs (most notably DCs) to Treg cell inhibition is also decreased (62, 88). In mice with collagen-induced arthritis (CIA), CD25^lo^Foxp3^+^CD4^+^ T cells are transformed into Th17 cells (arthritic synovial fibroblasts promote this transformation). These cells, called exFoxp3Th17 cells, accumulate in inflammatory joints and show a stronger ability to induce osteoclast production than any other T cell subset (89). Another characteristic of RA is the anoxic microenvironment of synovial tissue, neovascularization and cell exudation lead to synovial oxygen deficiency (90). During hypoxia, immune-inflammatory cells make adaptive response and activate pro-inflammatory signal pathways, and hypoxia-inducible factor-1α (HIF-1α) pathway is activated under hypoxia condition (90, 91). HIF-1α is expressed at high levels in synovial fibroblasts and macrophages from individuals with RA (92). HIF-1α can not only induce RORγt transcription to promote Th17 differentiation at the mRNA level, but also cooperate with RORγt protein to regulate downstream Th17 related genes. HIF-1α can also ubiquitinate and proteasome degradation by binding to Foxp3, resulting in the decrease of Foxp3 gene transcriptional activity and down-regulation of Foxp3 expression (93). Therefore, HIF-1α may be a potential target for RA therapy (94, 95). In addition, synovial fibroblasts (SFSs) also induce T cell differentiation in a hypoxic environment, resulting in a decrease in the number of Treg cells and an increase in the number of Th17 cells (96). Synovial hypoxia also changes the metabolic environment, while hypoxia also stimulates osteoclast-mediated bone resorption and aggravates joint injury (97). In addition, knock-out of the PD-1 gene in mice will cause a delayed in the development of specific autoimmune diseases, indicating that PD-1 plays a role in maintaining immune tolerance in immune regulation (98). Li et al. showed that the expression of PD-1 on the surface of CD4^+^ T and CD8^+^ T cells and the level of soluble PD-1 (soluble PD-1, sPD-1) in serum were significantly decreased in patients with RA (99). According to recent studies, T cells and pathogenic PD-1^+^ B cells accumulate in RA joints, and the expression of CXCR3 and GM-CSF in PD-1^+^ B cells is higher than in PD-1^-^ B cells (100).

## Treg Cells for Treatment of RA

Patients with autoimmune diseases often require lifelong immunotherapy, which is usually accompanied by serious adverse reactions and side effects. In recent years, the treatment of RA has gradually changed, and previous “step pyramid” treatment has been gradually replaced as guidelines have advocated the use of rheumatoid arthritis drugs such as disease-modifying anti-rheumatic drugs (DMARDs, methotrexate) at the early stage of the disease. For patients with a poor response to traditional DMARDs, biological DMARDs, such as TNF-α, CTLA-4 or small-molecule targeted DMARDs, such as the Janus kinase (JAK) inhibitor drugs facitinib and baracitinib, are recommended (4). Starting drug treatment in the early stage can effectively prevent the progression of the disease and reduce the rate of disease development. Because a large number of reports on the regenerative function of Treg cells have been published and the ideal treatment strategy is to induce self-tolerance before obvious tissue damage occurs, researchers have designed various strategies ways to increase the number of Treg cells and restore their function (101–107), by enhancing the function of Treg cells *in vivo*, including reducing the pro-inflammatory environment and enhancing the response of effector cells to inhibition (108–113) **(** **Table 2** **)**. Specific Treg cell-specific targeted gene proliferation stimulators were used to promote the expansion of Treg cells, or Treg cells were induced and expanded *in vitro* following the addition of immune complexes, and then injected into patients (114). The adoptive transfer of Treg cells increased the survival of Scurfy mice and prevented autoimmune diseases, and the removal of Treg cells before the disease increases the incidence and severity of the disease (115). At the same time, the transfer of Treg cells can slow the disease process, confirming that these cells have the potential to treat autoimmune diseases (116, 117). Adoptive cell therapy (ACT) uses Treg cells isolated from blood based on the cell surface labeling of CD4^+^CD25^+^CD127^-^ that are then expanded by treating them with anti-CD3, anti-CD28 and IL-2, followed by injection into the body (114). Expanded Treg cells have been used to treat a mouse autoimmune disease model before being used in the clinic. Early trials have been conducted in patients with type 1 diabetes and graft-versus-host disease after bone marrow transplantation showing a stable effect without serious adverse reactions (118–122). Models of CIA also showed inhibition, which significantly prevent the development of CIA (123). Importantly, when arthritis is inhibited in these models, not only are T and B cells inhibited by Treg cells but osteoclast-mediated bone destruction is also directly inhibited, preventing joint injury (124–127).

**Table 2:** The mechanisms by which the number or function of Treg cells is regulated.

Class	Mechanism underlying the effect	Effects on regulatory T cell	Ref.
**VIP**	Factors induce the inhibition of soluble protein secretion by increasing the expression of Foxp3 and TGF-β 1	Increases in the number and inhibitory activity of Treg cells changed the immune response to Th2 subsets	(100)
**Anti-TNF-a**	Induce Foxp3 expression	Increases in the number of circulating Treg cells	(101)
**CTLA-4-Ig**	Blocking T cell activation by binding to CD80/CD86 ligands	Induction of new iTreg cell populations Increase in the proportion of Treg cells Activate existing Treg cells	(102, 103)
**TGF**	Induction of Foxp3 expression	Induction of the differentiation of resistant Treg cells	(104)
**IL-2**	Activate the transcription factor STAT5	Promotes the activation and expansion of Treg cells	(105, 106)
**Rapamycin**	Blocking the AKT–mTOR-SMAD3 signaling axis Inducing Foxp3 expression	Inhibition of Teff cell proliferation Induction of the differentiation of Treg cells	(107)
**Anti-IL-6**	Rebalance the ratio of Foxp3/Ror-γt expression	Increases the Treg/Th17 ratio by suppressing Th17 generation	(108–110)
**IgD-Fc-Ig**	Restore the Th17/Treg cell subset balance	Reverse the imbalance of Th1/Th2 and Th17/Treg cell subsets	(111)
**Anti-IL-17**	Increase the Treg/Th17 ratio	Inhibition of the pro-inflammatory Th17 pathway	(112)

Ag, Antigen; iTreg, Induced Treg cell; Th, T helper cell.

Before the implementation of ACT, some key technical problems must be solved. Since Treg cells identify specific antigens, the first problem to be solved is the method used to isolate specific Treg cells *in vitro*. Both CD4^+^CD127^low/-^ and CD4^+^CD127^low/-^CD25^+^ T cells have been used for Treg amplification. The expansion of CD4^+^CD127^low/-^ cells requires the addition of rapamycin to maintain the purity of their lineage. CD4^+^CD127^low/-^CD25^+^ T cells, particularly the expansion of CD45RA^+^ subsets, produces a high yield of Treg cells that maintain high Foxp3 expression in the absence of rapamycin (128, 129). In the presence of anti-CD3/anti-CD28 and IL-2, this scheme can increase the number of cells up to thousands of times without losing the inhibitory activity of Treg cells (130). Since IL-2 is a key cytokine required for T cell activation and proliferation and nTregs express CD25 at high levels, they are highly sensitive to IL-2 stimulation. IL-2 (especially low-dose IL-2) preferentially amplifies Treg cells (131, 132). Although low-dose IL-2 directly increase the number of Treg cells *in vivo*, this effect is short-lived, once the treatment is stopped, the effect will be significantly reduced, and the effect of IL-2 itself on other effector cells must be considered.

The second problem is how to effectively expand antigen-specific Treg cells without losing their specificity or inhibitory function. Some studies have shown that amplified Treg cells tend to express IL-17, and CD4^+^CD25^+^Foxp3^+^ Tregs may be able to transform into pathogenic Th17 cells after repeated amplification (133–135). These studies show that the epigenetic stability of Tregs is unstable, and further studies have shown that the use of CD45RA^+^ as an additional marker for Treg isolation minimizes epigenetic instability due to amplification and avoids the increase in inflammation associated with Treg cell conversion into Th17 cells (135, 136).

The *in vivo* environment is complex, and *in vitro* cell therapy is inevitably time-consuming and expensive. Researchers have not clearly determined whether the expansion of Treg cells *in vivo* is better than *in vitro* expansion, and thus they have attempted to combine the two approaches, such as the application of autoantigen in an incomplete adjuvant (137), and the combination of tolDCs and Treg-induced peptide, which inactivate effector cells and promote the function of Treg cells (138).

## Stability of Treg Cells

Maintaining the stability and plasticity of Treg cells *in vivo* is one of the bottlenecks of ACT. Foxp3 is the main regulator of immunosuppression in Treg cells (139), and participates in the gene expression, function and survival of Treg cells. Its expression is regulated by transcriptional regulation, epigenetic regulation and post-translational regulation and is indispensable for the maintenance of immune self-tolerance (14, 140–142). The transcription factors NFAT, STAT5 and Foxo1 directly interact with the Foxp3 gene promoter to regulate the expression of Foxp3. The element of the conserved non-coding sequence (CNS) at the Foxp3 gene site regulates gene expression by recruiting transcription factors (143–145). The most noteworthy element is the methylation of CpG islets at the second intron enhancer site, also known as major TSDR(conserved non-coding sequence 2, CNS2), which is a region that is specifically demethylated in Treg cells. CNS2 demethylation stabilizes the expression of Foxp3 (146–148). Using CRISPR technology, recent studies have shown that ubiquitin-specific peptidase 22 (Usp22) is a positive regulator of stable Foxp3 expression, while ring finger protein 20 (Rnf20), an E3 ubiquitin ligase, is a negative regulator of Foxp3 (149). Phosphorylation, acetylation and ubiquitin are also considered factors regulating the stability of Foxp3 (145). Foxp3^+^nTreg cells are highly proliferative and highly stable. They may be able to recognize self-antigens or microbial antigens from symbiotic microorganisms (150–152).

In the past two years, researchers have performed numerous studies designed to improve the stability and optimize the function of Treg cells. For example, Chen *et al.* reported a more stable function for CD4 ^+^ CD126 ^low/-^ Foxp3^+^ cells than CD126^high^nTreg cells that remain stable even under inflammatory conditions (153). Human CD8^+^ regulatory T cells stimulated with rapamycin and TGF-β 1 also showed stable inhibitory ability in inflammatory environment (154). Park *et al.* found that daurinol, a natural arylnaphtholide isolated from the medicinal plant *Haplophyllumdauricum*, increases Treg cells stability by inducing DNA demethylation in the Foxp3 promoter region (155). PTPN2 promotes the stability of the Foxp3 mRNA in RORγt^+^ Treg cells, while the deletion or decreased expression of PTPN2 will promote the pathogenic transformation of Treg cells (156). TNFRII (TNF receptor type II) ^+^ Treg cells stably express Foxp3 through hypomethylation, and adoptive transfer of TNFRII^+^ Treg cells reduces the inflammatory response (157). The deletion of TNF receptor 2 causes Treg cells to show a Th17-like inflammatory phenotype (158). In addition, intracellular metabolic intermediates and environmental metabolites can also regulate the expression of Foxp3 in Treg cells (14). More interestingly, Treg cells have been modified to produce stable human Treg cells that target homing receptors, and these Treg cells migrate to specific sites or tissues to achieve more targeted immunosuppression and epigenetic stability under inflammatory conditions (159).

Genome editing technology is also used to enhance the stability of Treg cells. The expression of these genes is modified by CRISPR/Cas-9. Knockout of the PD-1 gene to modify T cells has been used in cancer therapy (160), which provides opportunities for the application of gene editing technology in Treg cells, such as knock-out of the genes that inhibit the function of Treg cells and up-regulation of the genes that can stabilize the expression of Foxp3. For example, knockout of the Stub1 gene increases the expression of Foxp3, and the up-regulation of CTLA-4, PD-1 and BACH2 will increase the stability of Treg cells (86). Using CRISPR technology, recent studies have shown that ubiquitin-specific peptidase 22 (Usp22) is a positive regulator of stable Foxp3 expression, while ring finger protein 20 (Rnf20), an E3 ubiquitin ligase, is a negative regulator of Foxp3 (149). In summary, CRISPR/Cas-9 technology is a new approach for RA therapy. The molecular mechanism of Foxp3 has been extensively studied. However, other studies have shown that the expression of Foxp3 alone is not sufficient to regulate gene expression in Treg cells (161–163). For example, an approximately 70% difference in the genomes of Foxp3^+^ nTreg cells and Foxp3-overexpressing Tconv cells has been identified, and the latter does not express some Treg signaling genes, such as Ikzf4 (Eos) and Ikzf2 (Helios) (163). However, the gene expression pattern of Foxp3-deleted Treg cells isolated from Foxp3 gene-deficient mice resembled Foxp3-intact normal nTreg cells (161, 164). The Treg-specific CpG demethylation (165) and histone modification also occurred before the expression of Foxp3 (166). Based on these findings, the cells that determine cell fate and differentiation develop long before Foxp3 expression. Therefore, the expression of Foxp3 alone does not represent the complete function of mature Treg cells. Foxp3 independent cell typing and immature tTreg cells differentiation indicate a Foxp3 independent genetic mechanism that controls the function and differentiation of early tTreg cells.

These Treg-specific genetic patterns are helpful for us understanding the function of Treg cells. The ideal strategy to stabilize the inhibitory function is to transform both initial and effector/memory Tconv cells into functionally stable Foxp3^+^ Treg cells. Foxp3 expression is induced in Tconv cells by treatments with targeting different signal transduction pathways, such as the TCR-DK8/19 pathway (167), AKT-mTOR pathway (168, 169), TGF-β-SMAD pathway (170). Continuous stimulation of Tconv cells by TCR might partially induce Treg-type DNA demethylation (171). Therefore, continuous TCR stimulation at the appropriate intensity likely induces the differentiation of developing T cells into functionally stable tTreg cells and the transformation from Tconv cells to Treg cells.

## Conclusions

Treg cells use multiple molecular mechanisms to inhibit the adverse reactions of RA, and their application may significantly control the progression of RA. Treg cells as intelligent “drugs” are attracting researchers’ interest. The successful application of Treg cell therapy in autoimmune diseases and transplantation will encourage clinical application of this method in the treatment of other non-immune diseases, such as tissue repair and neurological disorders (172). Although the molecular characteristics of human and mouse Treg cells are very similar, they are not the same, and the assessment of animal models *in vitro* is limited to a certain pathway. However, the *in vivo* environment is complex, and thus methods to better transform and apply the results obtained in animal experiments to human are worth examining. The identification of Treg cells *in vitro*, in addition to the use of more stringent markers, the determination of whether the drugs used for the treatment of RA disease will affect the phenotype of Treg cells, and an assessment of whether the function of Treg cells improves after treatment in a specific or non-specific manner is also worthy of further discussion. In addition, the time and stage of disease development are related to the therapeutic effect of ACT, and this treatment will be more effective in the early stage of the disease (173). Given the ability of Treg cells to specifically detect antigens through the TCR, developing an ACT that acts directly on a specific site or detects an antigenic site may be an ideal approach, Chimeric antigen receptor (CAR) Treg cells specifically migrate to target sites and show more obvious antigen-specific inhibitory activity (174).

In addition, in this new era of gene and cell therapy development, technological advances such as gene therapy-induced by target specificity or methods for delivering one or more genes to treat RA (175), single-cell transcriptome sRNA-seq has been used to analyze the expression of hundreds of genes in a single cell (176). The development of ATAC-seq has facilitated analyses of the occupancy of transcription factors in specific cell types (177). CRISPR/Cas-9mediated technology quickly and effectively generates genetic interference to identify and regulate the proliferation and function of T cells (178). All of these findings provide opportunities for the future development of accurate medical strategies and promote the clinical application of these treatments.

## Author Contributions 

QJ drafted the manuscript and designed the figures. GY and QL reviewed the manuscript structure and ideas. DC and SW conceived the topic and revised the manuscript. All authors contributed to the article and approved the submitted version.

## Funding 

This work was supported by the National Natural Science Foundation of China (Grant Nos. 81871709, 31711530025 and 81771759).

## Conflict of Interest 

The authors declare that the research was conducted in the absence of any commercial or financial relationships that could be construed as a potential conflict of interest.
